# Supplementary material for: Cortical circuits for cross-modal generalization
Source: Nat Commun. 2025 May 26;16:4230. doi: 10.1038/s41467-025-59342-9 (PMC12106601; doi:10.1038/s41467-025-59342-9)
Supplement: Supplementary file 1 — Supplementary Information [file 41467_2025_59342_MOESM1_ESM.pdf]

Supplementary Information for:

Cortical circuits for cross-modal generalization

Maëlle Guyoton[1]*, Giulio Matteucci[1]*, Charlie G. Foucher[1], Matthew P. Getz[2], Julijana Gjorgjieva[2] & Sami El-Boustani[1]

[1] Department of Basic Neurosciences, Faculty of Medicine, University of Geneva, 1 Rue Michel-Servet, 1206 Geneva, Switzerland.

[2] School of Life Sciences, Technical University of Munich, Maximus-von-Imhof-Forum 3, 85354 Freising, Germany.

* These authors contributed equally to this work.

Corresponding author: Sami El-Boustani (sami.el-boustani@unige.ch)

Supplementary Information

Supplementary Figures 1-15

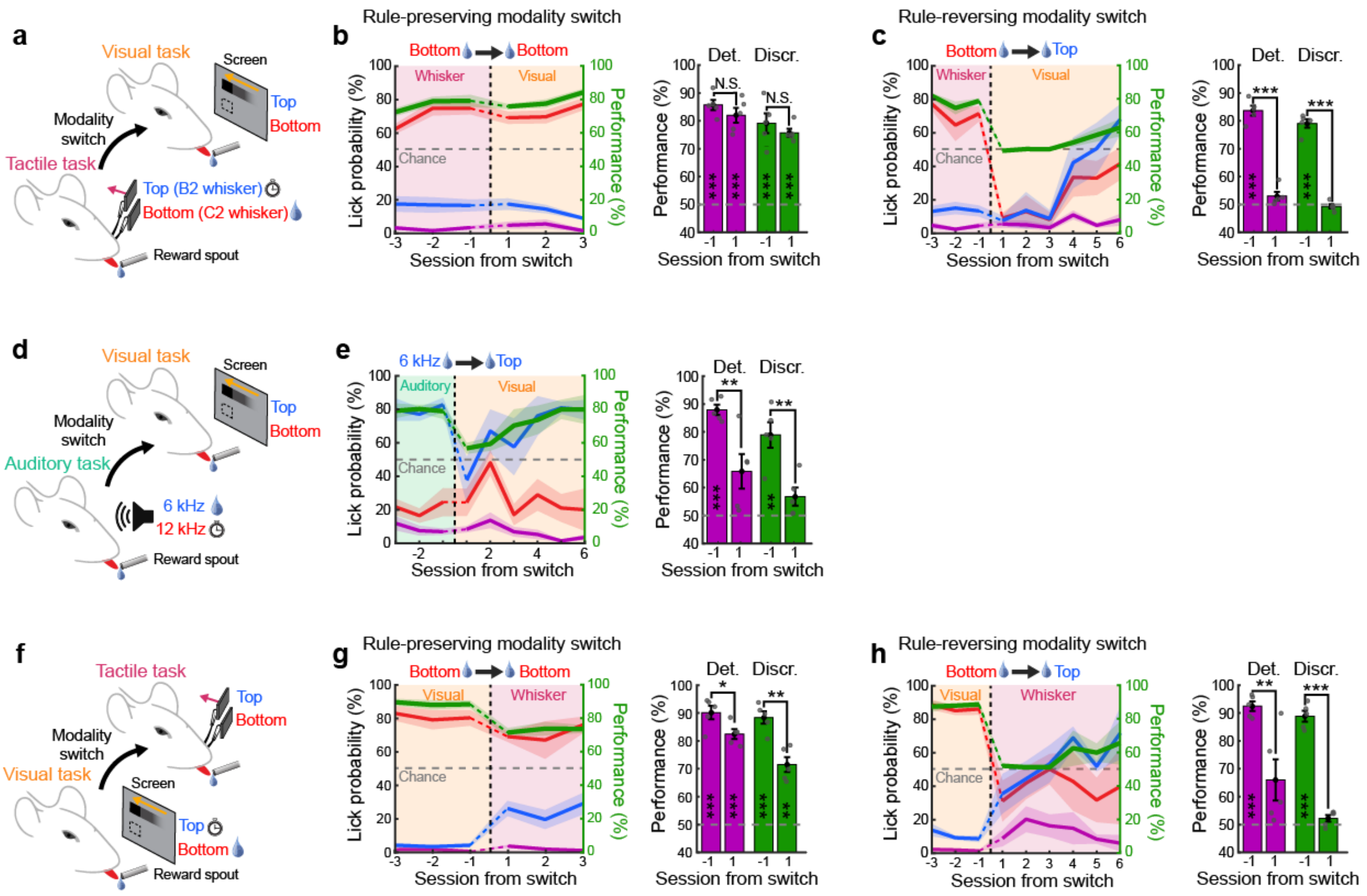

Supplementary Figure 1

Supplementary Fig. 1. Cross-modal generalization in mice across different conditions.

a, Schematic of behavioral paradigm where switches occur between a tactile task and a visual task. Bottom tactile stimuli were rewarded upon licking in the first task. **b,** Left: Average task performance and conditional lick probabilities across sessions for mice (N=5 mice) undergoing a rule-preserving modality switch (dashed vertical line). Shaded areas and color code as in Fig. 1e. Right: detection (purple) and discrimination (green) performance distribution for the session before and after the switch (two-sided paired t-test comparing days, Det. : N.S. $p=0.28$; Discr. : N.S. $p=0.3$). Performances are also tested against chance level (two-sided t-test, Det. : ***$p=4\times10^{-5}$ and ***$p=2.7\times10^{-4}$; Discr. : ***$p=0.001$ and ***$p=6.1\times10^{-5}$). Error bars: S.E.M. **c,** Same as panel b but for a rule-reversing modality switch (two-sided paired t-test comparing days, Det. : ***$p=1.1\times10^{-6}$; Discr. : ***$p=6.8\times10^{-5}$). Performances are also tested against chance level (two-sided t-test, Det. : ***$p=4.6\times10^{-5}$ and Blank $p=0.12$; Discr. : ***$p=4.5\times10^{-5}$ and Blank $p=0.41$). **d,** Schematic of behavioral paradigm where switches occur between an auditory discrimination task with two pure tones (6 kHz and 12 kHz) and a visual task. The 6 kHz tone is always associated with a water reward. **e,** Same as panel b for an auditory to visual modality switch, where the top visual stimulus is associated to a reward (N=5 mice, two-sided paired t-test comparing days, Det. : **$p=0.009$; Discr. : **$p=0.01$). Performances are also tested against chance level (two-sided t-test, Det. : ***$p=3.1\times10^{-5}$ and Blank $p=0.063$; Discr. : **$p=0.003$ and Blank $p=0.1$). **f,** Schematic of behavioral paradigm where switches occur between a visual task and a tactile task with the bottom visual stimulus being the rewarded one. **g,** Same as panel b for mice undergoing a rule-preserving switch (N=5 mice, two-sided paired t-test comparing days, Det. : *$p=0.033$; Discr. : **$p=0.003$). Performances are also tested against chance level (two-sided t-test, Det. : ***$p=7.4\times10^{-5}$ and ***$p=5.6\times10^{-5}$; Discr. : ***$p=6.7\times10^{-5}$ and **$p=1.3\times10^{-3}$). **h,** Same as panel g but for rule-reversing modality switches (N=5 mice, two-sided paired t-test comparing days, Det. : **$p=0.008$; Discr. : ***$p=2.5\times10^{-4}$). Performances are also tested against chance level (two-sided t-test, Det. : ***$p=1.6\times10^{-5}$ and Blank $p=0.096$; Discr. : ***$p=4\times10^{-5}$ and Blank $p=0.14$). For cases with spatial rule-reversing modality switch or in absence of spatial prior (i.e. panels c,e,h), behavioral curves are shown for 6 consecutive sessions following the switch.

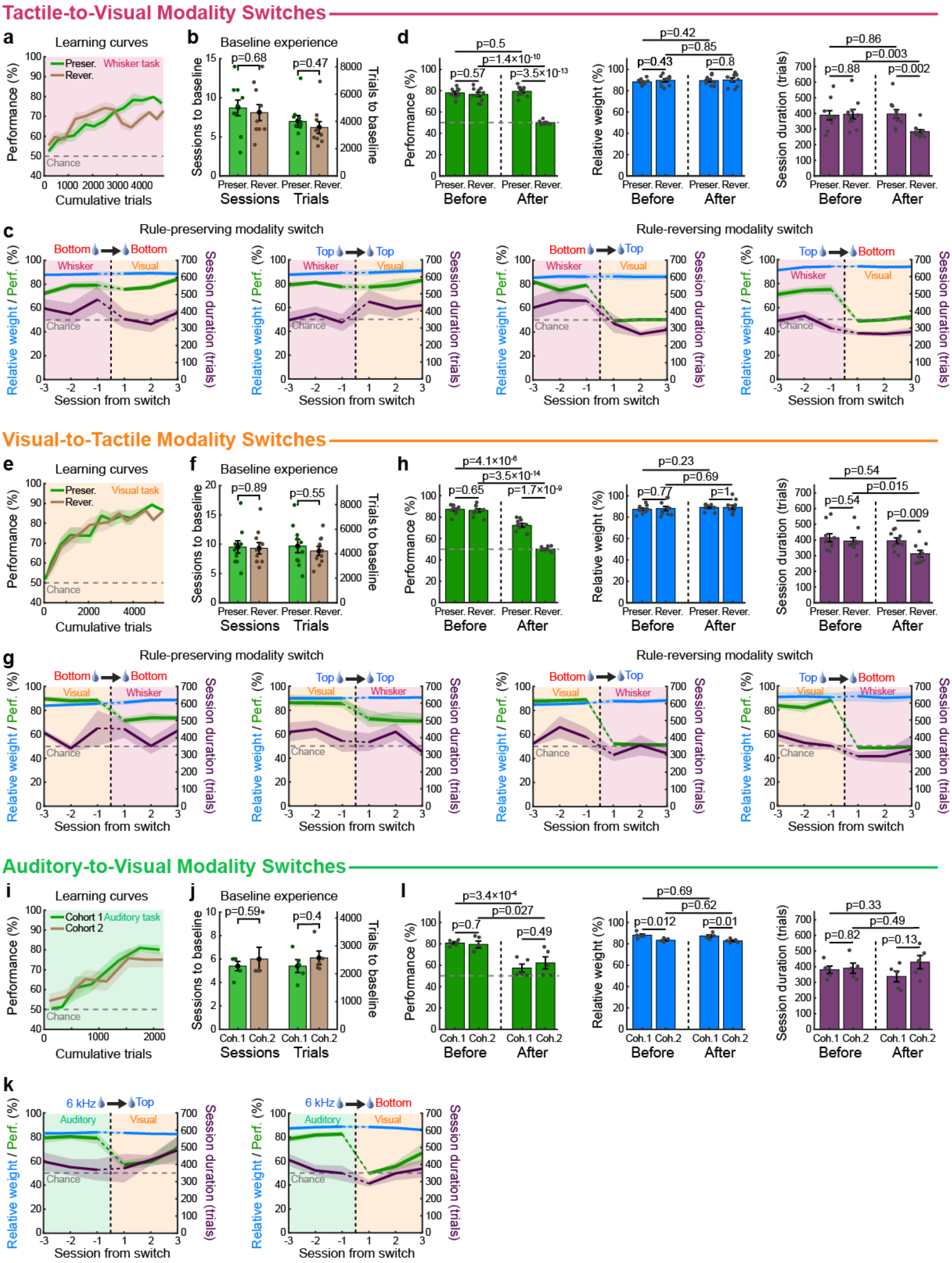

Supplementary Figure 2

Supplementary Fig. 2. Learning trajectory, task experience, task performance and motivational state in the different cohorts of mice and different tasks.

a, Discrimination learning curves for the cohorts of mice performing the whisker task before rule-preserving (green, N=10 mice) or rule-reversing (brown, N=10 mice) modality switch to a visual task. Shaded areas: S.E.M. **b,** Comparison of number of sessions and trials experienced by mice in the rule-preserving and rule-reversing cohorts before the switch (unpaired two-sided t-test). **c,** Switch-aligned task performance (green), relative weight (blue) and session duration (purple) averaged across mice for all tactile-to-visual conditions. Shaded areas: S.E.M. **d,** Comparison of performance (green), relative weight (blue) and session duration (purple) averaged over the 3 days preceding (before) or following (after) modality switch (unpaired two-sided t-test). In all plots, horizontal gray dashed line indicates performance chance level and vertical black dashed line indicates the switch. **e-h,** same as panels a-d but for cohorts of mice performing the visual task before rule-preserving (green, N=10 mice) or rule-reversing (brown, N=10 mice) modality switch to the whisker task. **i-l,** Same as panels a-d but for two cohorts of mice (N=5 mice each) performing the auditory task before modality switch to the visual task. All bar plots indicate the average value and S.E.M.

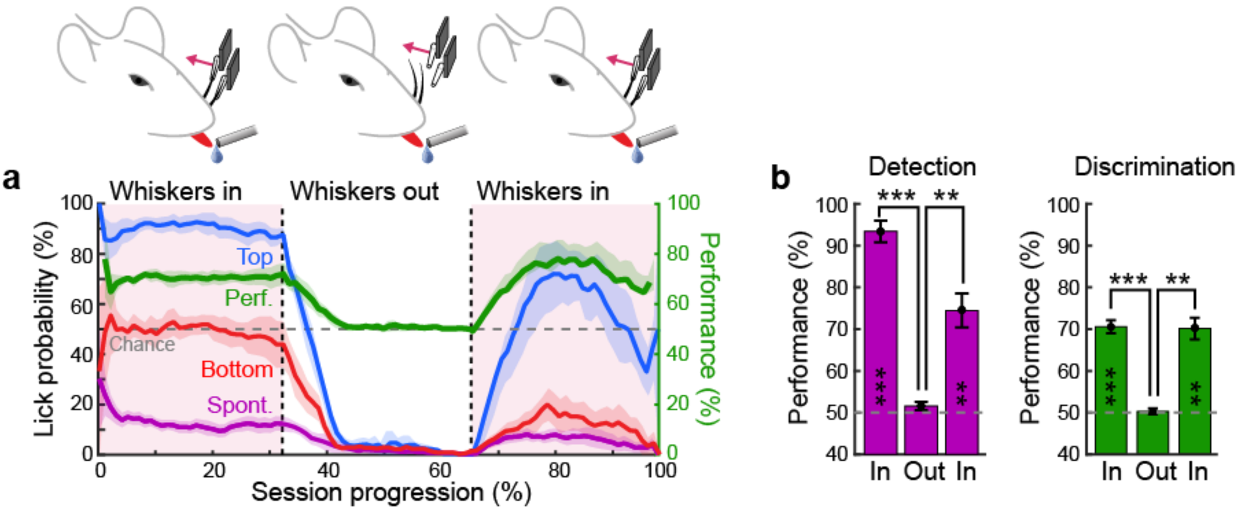

Supplementary Figure 3

Supplementary Fig. 3. Performance of mice at the whisker discrimination task with and without whiskers in the piezo stimulators.

a, Conditional lick probabilities and performance during the whisker discrimination task averaged across expert mice (N=5 mice). Sessions were normalized between beginning (0%) and end (100%) before the average was performed. Color code is the same as in Fig. 1. During these sessions, mice performed the task for approximately one-third of the trials with whiskers present in the capillary tubes used to deliver tactile stimulations. Subsequently, the whiskers were removed while maintaining the tube positions, and the task continued for an equivalent duration. Finally, the whiskers were reinserted to confirm that mice were still motivated to perform the task. **b,** Comparison of detection (purple) and discrimination (green) performance during the 3 different phases of the sessions described in panel a. Comparisons are performed between consecutive phases (paired two-sided t-test, Detection: ***$p=3\times10^{-5}$ and **$p=0.004$, Discrimination: ***$p=1.1\times10^{-4}$ and **$p=0.008$) and with chance level (two-sided t-test, Detection: ***$p=7.7\times10^{-5}$, Blank $p=0.19$, **$p=0.004$; Discrimination: ***$p=1.9\times10^{-4}$, Blank $p=0.71$, **$p=1.5\times10^{-3}$). Mice were not able to perform the task in absence of direct whisker stimulations. Error bars: S.E.M.

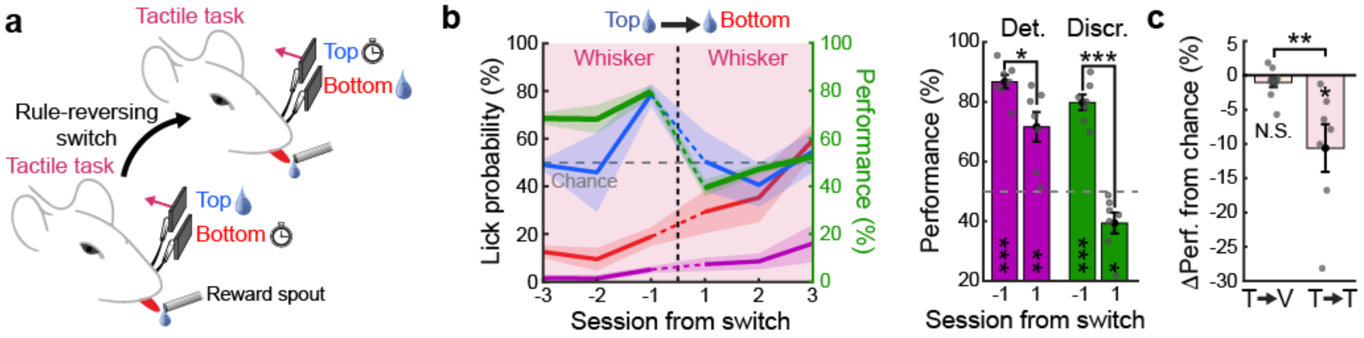

Supplementary Figure 4

Supplementary Fig. 4. Relearning the whisker discrimination task following a reversal of the spatial rule within the same modality.

a, Schematic of the behavioral paradigm for rule-reversing switch within the tactile modality. Once mice became experts in the whisker discrimination task, the spatial rule of the task was reversed. **b,** Left: Average task performance across sessions for mice exposed to the reversed reward association (N=7 mice). Shaded area: S.E.M. Vertical dashed line indicates the transition from one rule to the other. Right: detection (purple) and discrimination (green) performance distribution for the session before and after the switch (two-sided paired t-test comparing days, Det. : *p=0.017; Discr. : ***$p=8.3\times10^{-5}$). Performances are also tested against chance level (two-sided t-test, Det. : ***$p=3.8\times10^{-6}$ and **p=0.005; Discr. : ***$p=2.5\times10^{-5}$ and *p=0.022). Error bars: S.E.M. **c,** Comparison of performance change relative to chance level after rule switch across sensory modalities (i.e. tactile to visual) or within the whisker task (N=10 mice for visuo-tactile and N=7 mice for tactile only, unpaired two-sided t-test, **p=0.0056). Error bars: S.E.M. Mice maintained their responses to whisker stimulations significantly above chance level, yet their performance on the discrimination task fell significantly below chance (two-sided t-test, *p=0.022, N.S. p=0.15). This suggests a reduced adaptability to a reversal of the spatial rule within the same modality, potentially due to a stronger habitual response linked to the previously rewarded whisker or an entrenched bias regarding the saliency of whisker stimulations.

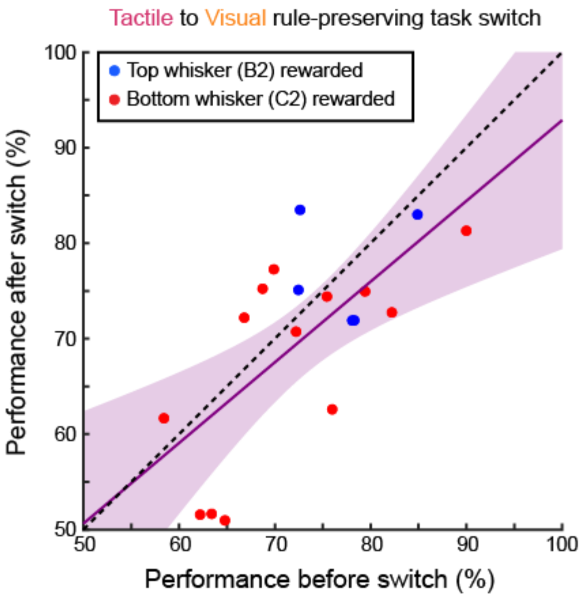

Supplementary Figure 5

Supplementary Fig. 5. Performance level is maintained after switches from the whisker discrimination task to the visual task.

Comparison between performance in the whisker task the session before the switch and the performance in the visual task the session following the switch. This includes mice that experienced the switch, showcasing varying performance levels and rewarding either the top whisker (blue) or the bottom whisker (red). Performances before and after the switch were correlated (N=18 mice, Pearson coefficient: 0.68, p=0.0018) and close to the unity dashed line. Shaded area: 95% confidence interval. Mice exhibit consistent discrimination performance following modality switch, suggesting that cross-modal generalization may correspond to the initial sensorimotor learning level, or that shared influences, such as thirst-related motivation, impact both tasks equally.

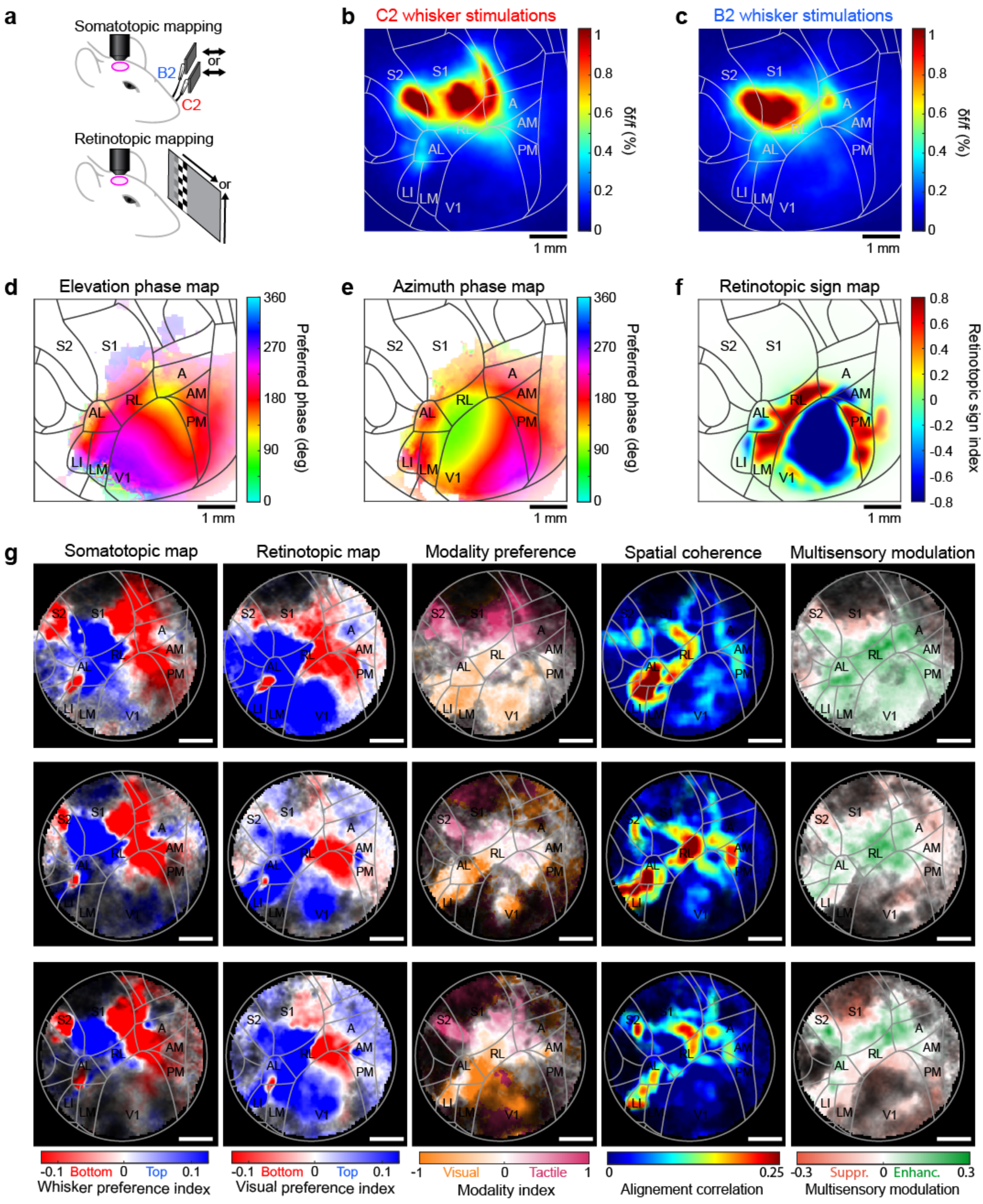

Supplementary Figure 6

Supplementary Fig. 6. Average whisker and retinotopic maps with examples of single mouse visuo-tactile functional maps obtained in wide-field imaging.

a, Schematic of the protocols used to measure single whisker response maps (top) and retinotopic maps (bottom) in transgenic mice expressing GCaMP6f in layer 2/3 during wide-field imaging through a cranial window. **b,** Response map averaged across mice (N=41 mice) for C2 whisker stimulations. Projection of the Allen Mouse Brain Atlas is overlaid on top with areas names for reference. **c,** Same as in panel b for B2 whisker stimulations. **d,** Elevation phase map averaged over mice (N=50 mice). Pixels with low response amplitude are displayed in white. **e,** Azimuth phase map average over the same mice as in panel d. **f,** Retinotopic sign map computed based on the elevation and azimuth phase maps of panels d and e. **g,** Representative examples of visuo-tactile functional maps obtained from sparse noise protocol responses from 3 different mice (rows). From left to right: 1. Whisker preference map used to describe somatotopy in the vertical space with red colors indicating preference for the bottom whisker and blue colors indicating preference for the top whisker. 2. Visual position preference map used to describe retinotopy in vertical space. 3. Modality preference map describing the dominant modality for each pixel. 4. Spatial coherence map between retinotopic and somatotopic maps. 5. Multisensory modulation map indicating areas with responses enhancement or suppression for visuo-tactile stimuli.

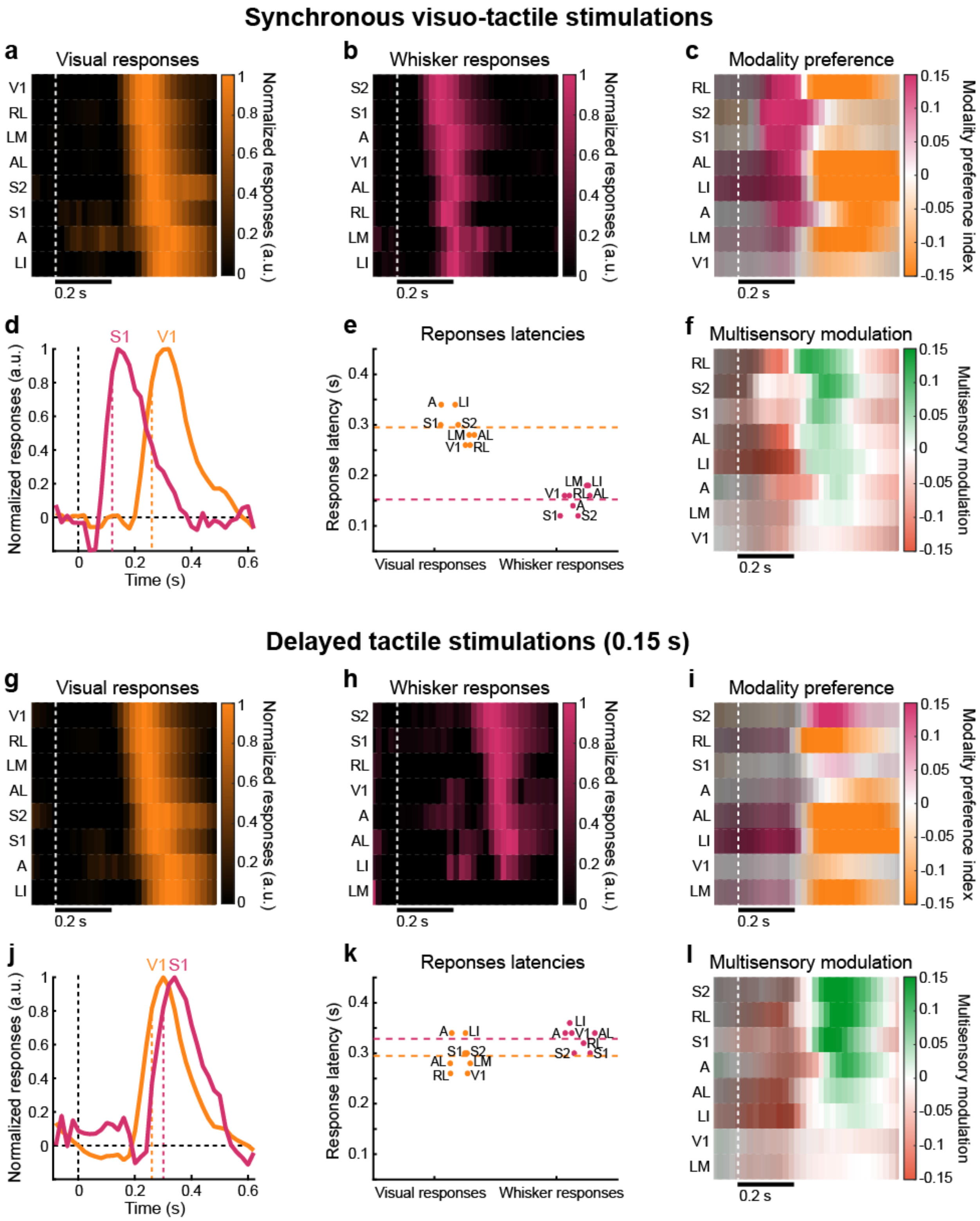

Supplementary Figure 7

Supplementary Fig. 7. Comparison of cortical response dynamics for synchronous or delayed visuo-tactile stimulations.

a, Average activation time course for each visually responsive area following stimulus onset (N=29 mice). Areas are ordered based on response onsets. White dashed line indicates stimulus onset. **b,** Same as panel a for responses in cortical areas evoked by whisker stimuli. **c,** Time course of the modality preference index across cortical areas with visuo-tactile responses. **d,** Average response time course in the primary visual cortex (V1) and the whisker primary somatosensory cortex (S1) highlighting an offset of about 0.15 s between cortical responses evoked by the two modalities. Colored dashed lines indicate response latency computed as the time point where the response curve reaches 80% of its peak amplitude. **e,** Distribution of response latencies for cortical areas with evoked visual (orange) or whisker tactile (magenta) responses. Colored dashed lines indicate the average latencies across areas. **f,** Time course of the multisensory modulation index across cortical areas with visuo-tactile responses. **g-l** Same as panels a-f for tactile stimuli presented with a delay of 0.15 s with respect to the visual stimuli to match response latencies in the cortical areas. Multisensory enhancement was observed when stimuli were synchronized or delayed though they were stronger when cortical responses had comparable latencies.

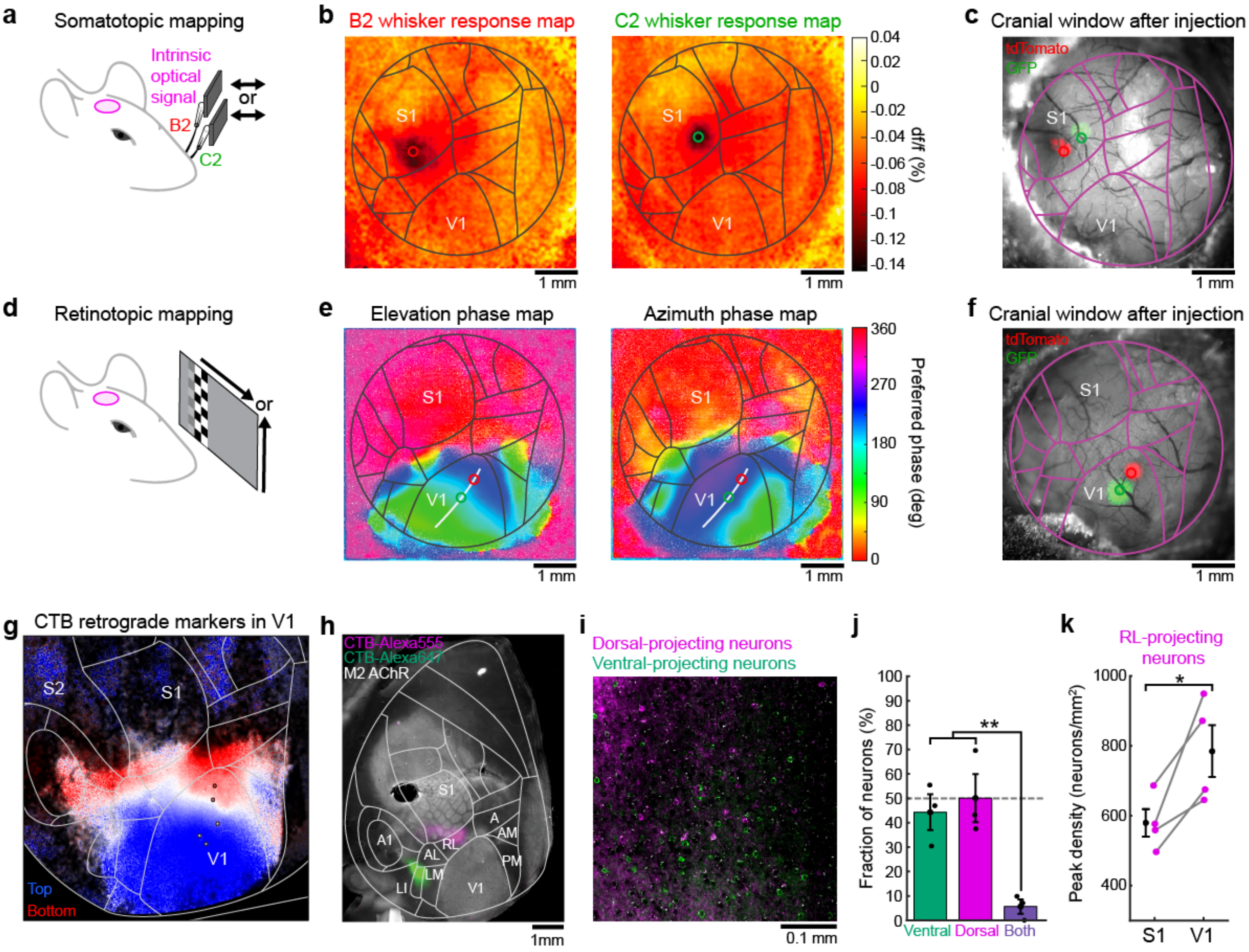

Supplementary Figure 8

Supplementary Fig. 8. Functional mapping of vertical positions in the visual or whisker somatosensory cortex for anatomical tracing.

a, Schematic of the protocol for mapping whisker-responding areas in the dorsal cortex. Single whiskers are stimulated repeatedly during intrinsic optical signal imaging. **b,** Change in red light reflectance on the surface of the cortex measured after averaging several trials of whisker stimulations relative to baseline. The left image reveals the barrel location corresponding to B2 whisker stimulations and the right plot for C2 whisker stimulations. **c,** Image of the cranial window several weeks after injection of viral vectors to express GFP and tdTomato in two distinct parts of the whisker somatotopic map. Locations of B2 and C2 barrels in S1 defined in panel b are indicated with circles overlaid on top of the fluorescent signal where injections were performed. **d,** Schematic of the protocol for retinotopic mapping in areas of the dorsal cortex. Horizontal or vertical bars drift across the screen and they are textured with a flickering checkerboard pattern to elicit strong cortical responses at different phases corresponding to the retinotopic preference. **e,** Retinotopic phase maps for elevation and azimuth on the dorsal cortex. These maps are then used to identify a gradient across vertical dimension in an iso-profile along the azimuth dimension. Two retinotopically distinct locations along the vertical axis are identified and labeled for viral injection. **f,** Image of the cranial window four weeks after injection of viral vectors to express GFP and tdTomato in two distinct parts of the primary visual cortex retinotopic map. Locations where stereotaxic injections were performed corresponding to panel e are indicated with circles overlaid on top of the fluorescent signals. **g,** Cholera Toxin B subunit (CTB) conjugated to different Alexa dyes was injected in different locations of the retinotopic map in V1 (circles). After perfusing the brain and flattening the cortex, a map was reconstructed over the dorsal cortex combining all injection sites to reconstruct areas where neurons project to the region of V1 encoding stimuli on the top (blue) or bottom (red) part of the visual field. All cortices with CTB labels were registered to a common atlas before performing the analysis (5 injection sites from N=3 mice). A similar analysis was performed previously with coronal slices[29]. **h,** Flattened cortex with injections of CTB conjugated with two different dyes (Alexa555 and Alexa647) in the dorsal and ventral visuo-tactile area, respectively. Immunohistochemical localization of M2 AChR is used to identify V1 and the barrels in S1 for atlas registration. **i,** Example image obtained with confocal microscopy where

dorsal-projecting neurons (magenta) and ventral-projecting neurons (green) are intermingled in V1. **j,** Fraction of neurons that projected to the ventral area exclusively (green), to the dorsal area exclusively (magenta) or to both (purple) (N=3 mice, unpaired two-sided t-test, **$p=1.8\times10^{-3}$, n=230 neurons for ventral, n=277 neurons for dorsal and n=26 neurons for both out of 26 fields-of-view). Neurons that project to the dorsal and ventral visuo-tactile areas display minimal overlap, suggesting they may process distinct types of visual information. **k,** Comparison of peak density of RL-projecting neurons in V1 and S1 as measured with confocal imaging (N=4 mice, paired two-sided Wilcoxon test, *p=0.042). Error bars: S.E.M.

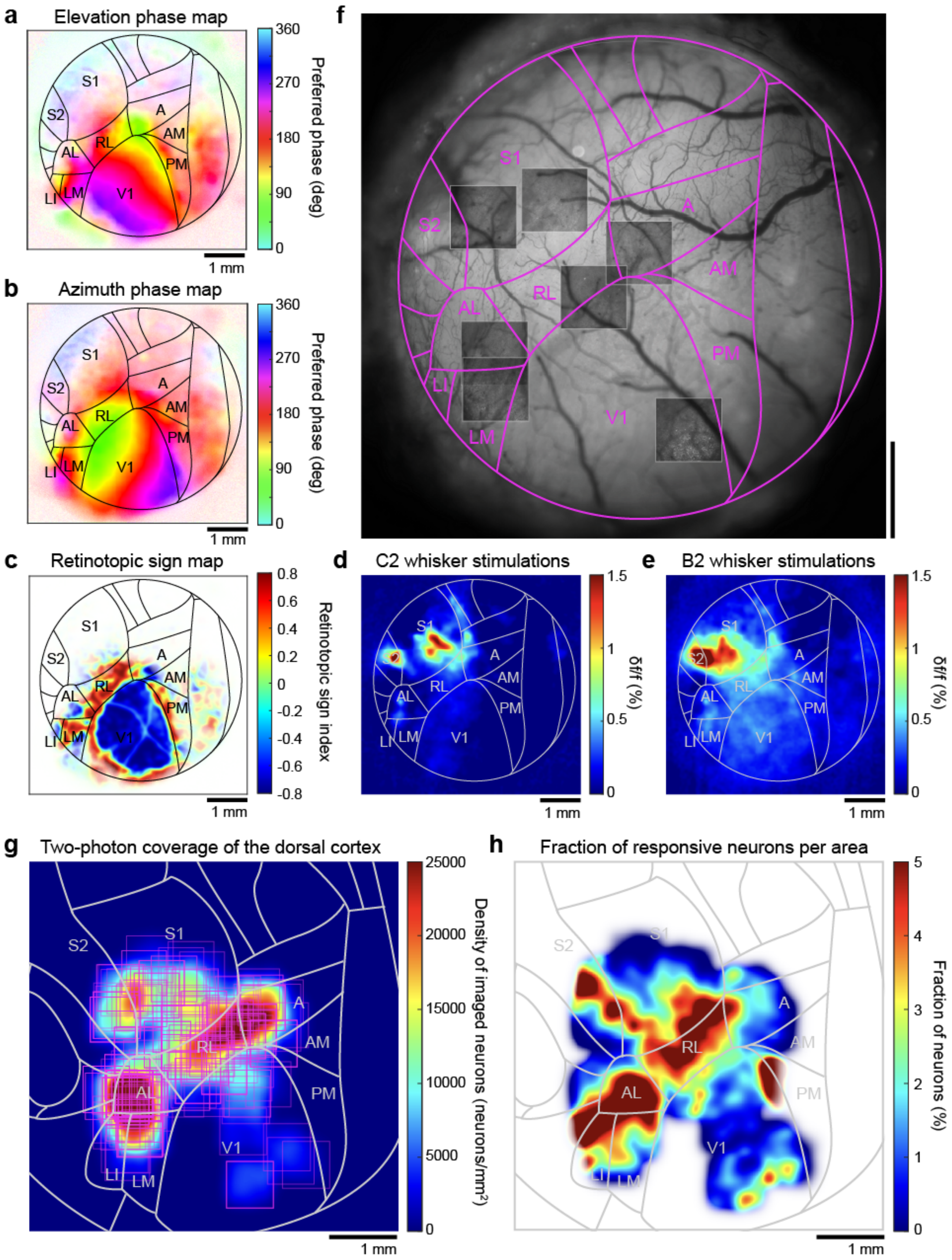

Supplementary Figure 9

Supplementary Fig. 9. Coverage of large portions of the cranial window for characterizing visuo-tactile responses at single-cell level using two-photon microscopy.

a-e, Functional maps are obtained and aligned with the Allen Mouse Brain Atlas for each individual mouse, ensuring precise localization of neurons within their respective areas. Subsequently, all maps are realigned to a common reference atlas to reconstruct wide-field maps. Visual stimulation protocols are used to obtain phase maps for elevation (panel a) and azimuth (panel b) to compute the retinotopic sign map (panel c). Additionally, whisker stimulations provide response maps for C2 (panel d) and B2 (panel e) whisker stimulations. **f,** Image obtained with wide-field imaging where blood vessels are well distinguished on the surface of the cortex. The atlas fitted with functional maps is overlaid on top in purple. For this mouse, seven fields-of-view were imaged using two-photon microscopy and positioned on the cortical surface by matching blood vessel patterns. **g,** Coverage of visual and tactile areas of the dorsal cortex with two-photon imaging across mice. Using the common atlas and information about all neuron's location, we can recreate a large-scale population using single-cell information. We could collect N=272 fields-of-view across N=25 mice. **h,** Fraction of neurons imaged with two-photon microscopy that were responsive to at least one stimulus condition. The areas of high responsivity are in accordance with wide-field imaging. White color indicates regions where no imaging was performed.

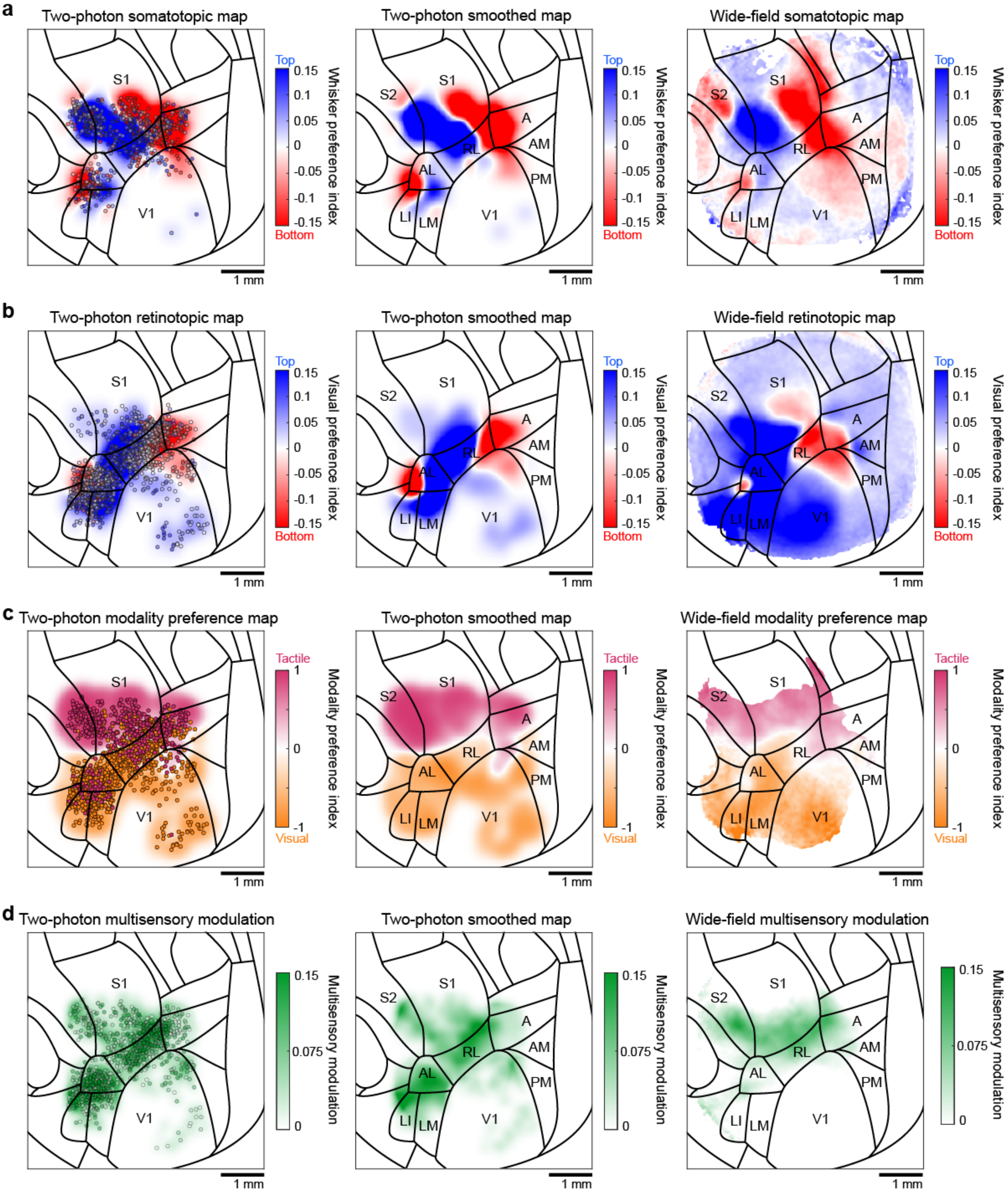

Supplementary Figure 10

Supplementary Fig. 10. Functional maps correspondence between wide-field and two-photon calcium imaging.

a, Left: Distribution of neurons imaged with two-photon microscopy with significant whisker tactile responses. Neurons are displayed on the common atlas and are color-coded based on their whisker preference (n=790 neurons). Middle: Large-scale functional map reconstructed from single neuron whisker preferences. Right: Somatotopic map for the whisker preference obtained with wide-field imaging. Pearson coefficient of correlation between the wide-field map and the two-photon reconstructed map: 0.847. **b,** Same as in panel a for neurons with significant visual responses (n=1,816 neurons, Pearson coefficient of correlation: 0.651). **c,** Same as in panel a displaying modality preference for all neurons with significant whisker tactile or visual responses (n=2,160 neurons, Pearson coefficient of correlation: 0.795). **d,** Same as in panel a displaying multisensory enhancement for all neurons with significant multisensory modulation. For this analysis, the multisensory modulation is computed as the variation between the most suppressed and most enhanced response across conditions for all neurons (n=2,497 neurons, Pearson coefficient of correlation: 0.4458). These neurons were from the same dataset used for Figure 5 (N=25 mice).

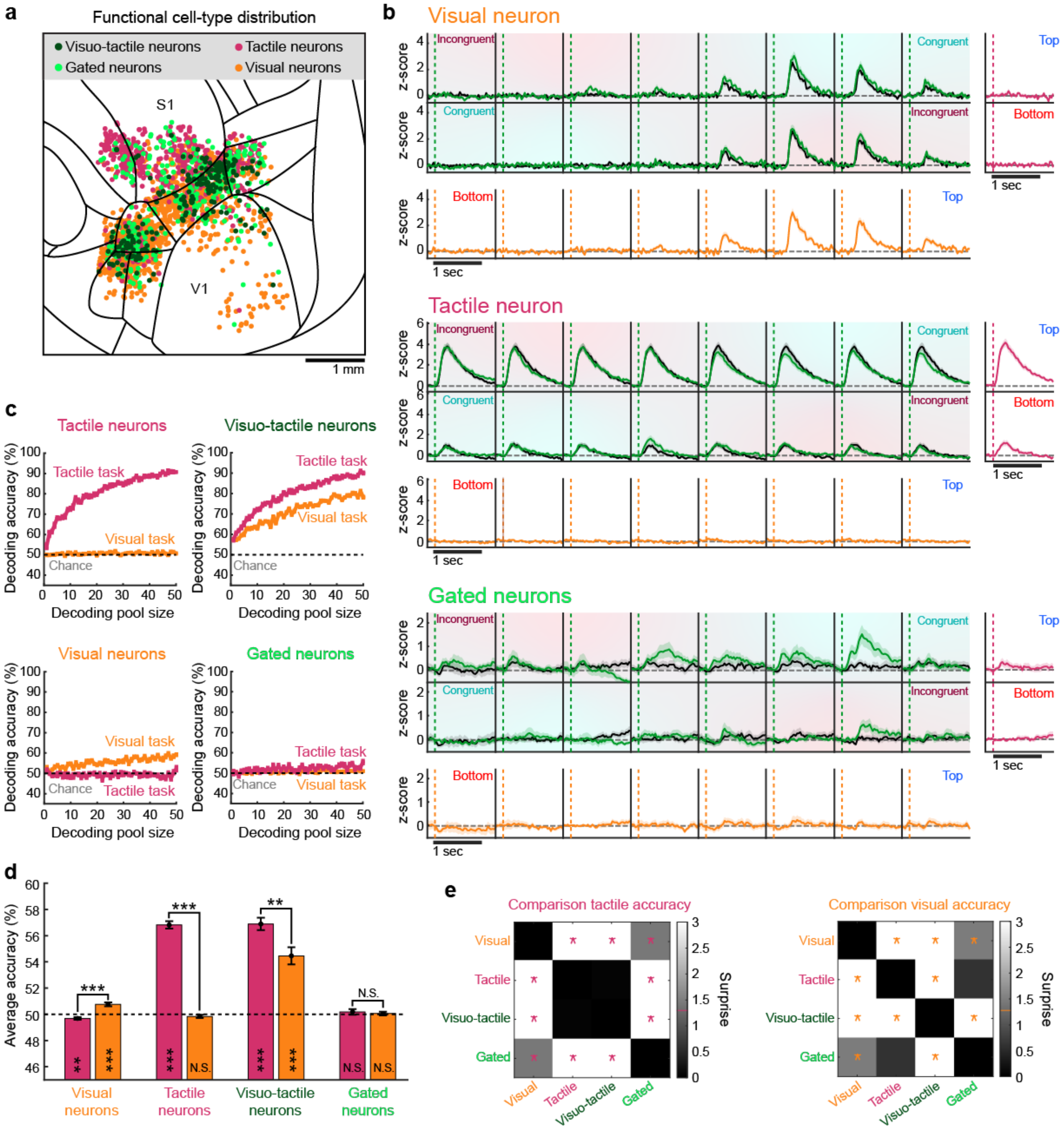

Supplementary Figure 11

Supplementary Fig. 11. Distribution of functional cell-types across the dorsal cortex and their impact on cross-modal generalization.

a, Distribution of significantly responsive neurons across the dorsal surface of the cortex (from the same N=25 mice presented in Figure 5). Common atlas overlaid for reference. Color code indicates functional cell-type of each neuron (n=2,703 neurons). Neurons were classified in four categories: tactile neurons (magenta) responded only to whisker stimulations (n=567); visual neurons (orange) responded only to visual stimulations (n=1,593); visuo-tactile neurons (dark green) responded to both visual and whisker stimulations (n=213); gated neurons (light green) responded only to concomitant visuo-tactile stimulations (n=319). **b,** Example visuo-tactile receptive field for a visual neuron (top), a tactile neuron (middle) and a gated neuron (bottom). Color code is identical to Fig. 5b. Note that the gated neuron responds only when visual and whisker stimulations are presented together in the top location (spatially congruent condition). **c,** Decoding accuracy for the tactile task and the visual task as a function of the pool size. A Bayesian decoder was trained to discriminate the location of whisker stimulation (magenta) based on the neuronal responses of a pool of neurons. The same decoder was then used to decode the location of visual stimuli (orange), mimicking the modality switch experienced by mice in the behavioral experiment. Error bars: S.E.M. Only visuo-tactile neurons were able to achieve good generalization performance across modalities because of their functional properties. Tactile neurons could only afford good decoding performance in the whisker-based task but could not maintain it when switching to the visual task. Performance chance level is indicated with a gray dashed line at 50%. **d,** Comparison of single-neuron decoding accuracy distributions on tactile responses (magenta) and generalization to the visual ones (orange) for each functional cell-type class (paired two-sided t-test comparing tactile and visual task, Visual: ***$p=9.4\times10^{-9}$, Tactile: ***$p=3.4\times10^{-94}$, Visuo-tactile: **$p=0.0034$, Gated: N.S. $p=0.56$). Decoding accuracies are also tested against chance level (two-sided t-test, Visual: **$p=0.004$ and ***$p=5.7\times10^{-7}$, Tactile: ***$p=6.8\times10^{-104}$ and N.S. $p=0.17$, Visuo-tactile: ***$p=7.6\times10^{-38}$ and ***$p=4.8\times10^{-11}$, Gated: N.S. $p=0.33$ and N.S. $p=0.69$). Error bars: S.E.M. **e,** Surprise matrix corresponding to all two-sided t-tests performed across tactile (left) or visual (right) conditions for data in panel d. Asterisks indicates surprise values larger than 1.301 corresponding to $p<0.05$.

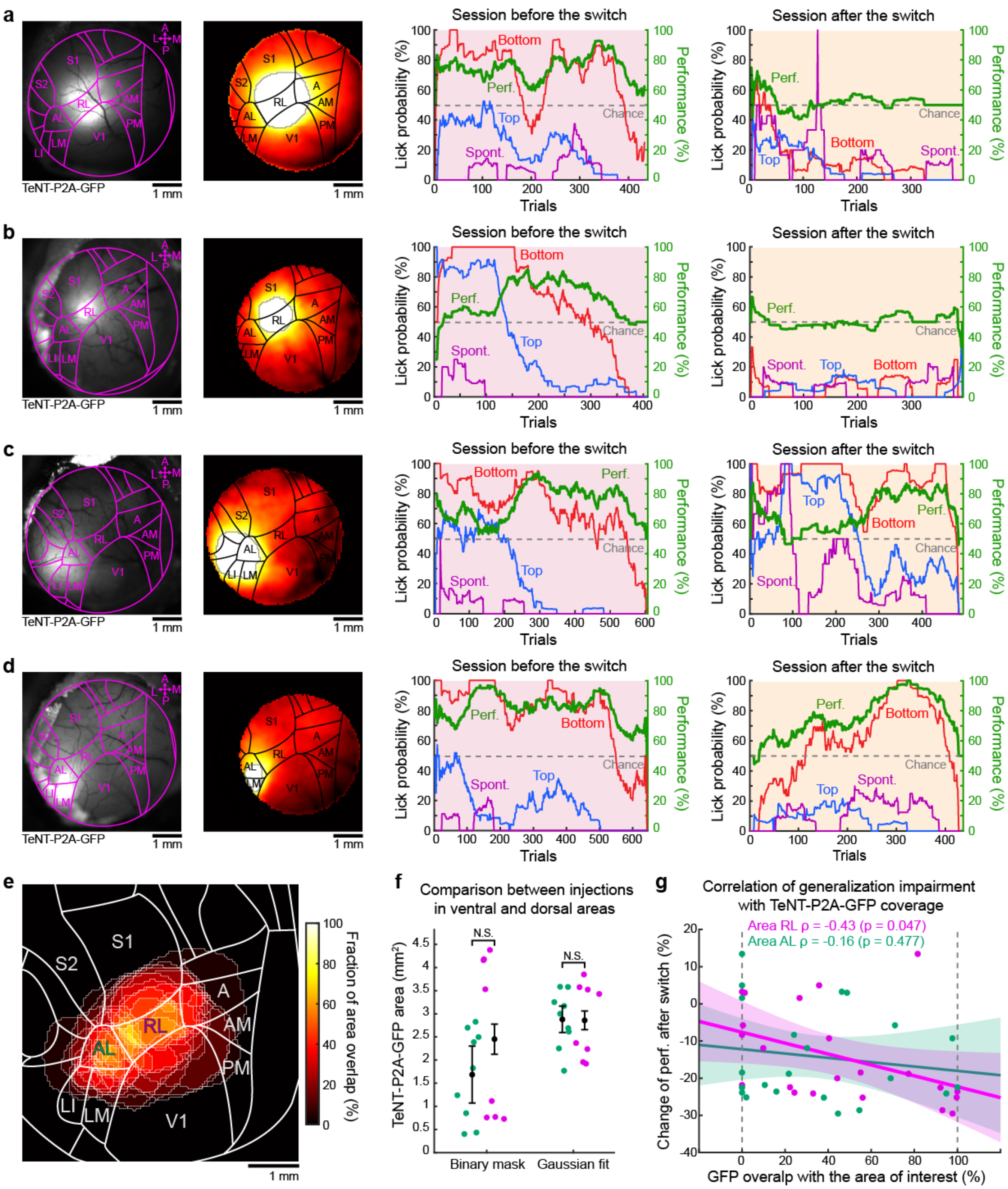

Supplementary Figure 12

Supplementary Fig. 12. Cross-modal generalization impairment following rule-preserving switches in relation to TeNT expression in different associative areas of the dorsal cortex.

a, Left: Example of TeNT-P2A-GFP expression pattern over the cranial window and corresponding estimate of expression coverage after blood vessels subtraction. Right: Effect of cortical silencing on cross-modal generalization from the tactile task to the visual task while preserving the spatial rule of the task. The discrimination performance dropped at chance level after the switch. **b-d,** Same as in panel a but for other example mice with cortical injections at various locations and the resulting impact on cross-modal generalization performance. Note that equal volumes of viral vectors were injected in each mouse; however, some expression patterns appear truncated at the edge of the window. **e,** Overall coverage across all experiments performed with this protocol. **f,** Comparison of estimated surface expressing TeNT-P2A-GFP between injections performed in ventral (green, N=9 mice) or dorsal (magenta, N=8 mice) areas. On the left, the surface was estimated using a binary mask obtained with the normalized fluorescent signal (unpaired two-sided t-test, p=0.15). On the right, the surface of expression was estimated using a Gaussian fit to include regions obstructed by the dental cement at the edge of the cranial window (see Methods, unpaired two-sided t-test, p=0.66). Error bars: S.E.M. **g,** Example of correlation between the level of TeNT-P2A-GFP overlaps with a specific area (here RL or AL) and the corresponding drop of discrimination performance after switch. To obtain a more robust effect for the correlation, we computed the performance changes over 3 consecutive sessions after switch (N=22 mice, Pearson coefficient: -0.43, p=0.047 for RL; Pearson coefficient: -0.16, p=0.48 for AL).

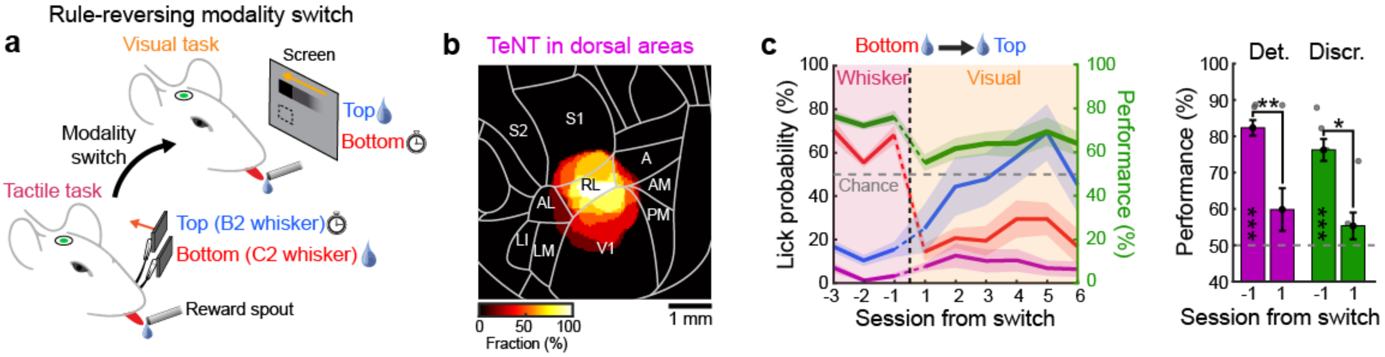

Supplementary Figure 13

Supplementary Fig. 13. Relearning after rule-reversing modality switch in mice expressing TeNT-P2A-GFP in the dorsal area RL.

a, Schematic of behavioral paradigm where the modality switches occur between a tactile task and a visual task in mice expressing TeNT-P2A-GFP in RL. The bottom whisker stimulus is associated with the reward initially while the top visual stimulus becomes associated to rewards after the modality switch. **b,** Average TeNT-P2A-GFP coverage in all mice where dorsal neurons were silenced (N=6 mice). **c,** Left: Average task performance and conditional lick probabilities across sessions for mice population in panel b (N=6 mice) switching from the tactile task to the visual task with a spatial rule reversal. Shaded area: S.E.M. Black dashed line indicates the switch between modalities. Right: detection (purple) and discrimination (green) performance distribution for the session before and after the switch (two-sided paired t-test comparing days, Det. : **p=0.005; Discr. : *p=0.016). Performances are also tested against chance level (two-sided t-test, Det. : ***$p=2.5\times10^{-5}$ and Blank p=0.15; Discr. : ***$p=3.4\times10^{-4}$ and Blank p=0.21). Error bars: S.E.M. Contrary to mice with intact cortical activity, mice with silenced RL area relearned the visual task faster after the rule-reversing modality switch, indicating a lack of prior on the spatial rule in these mice despite their previous experience with the tactile task.

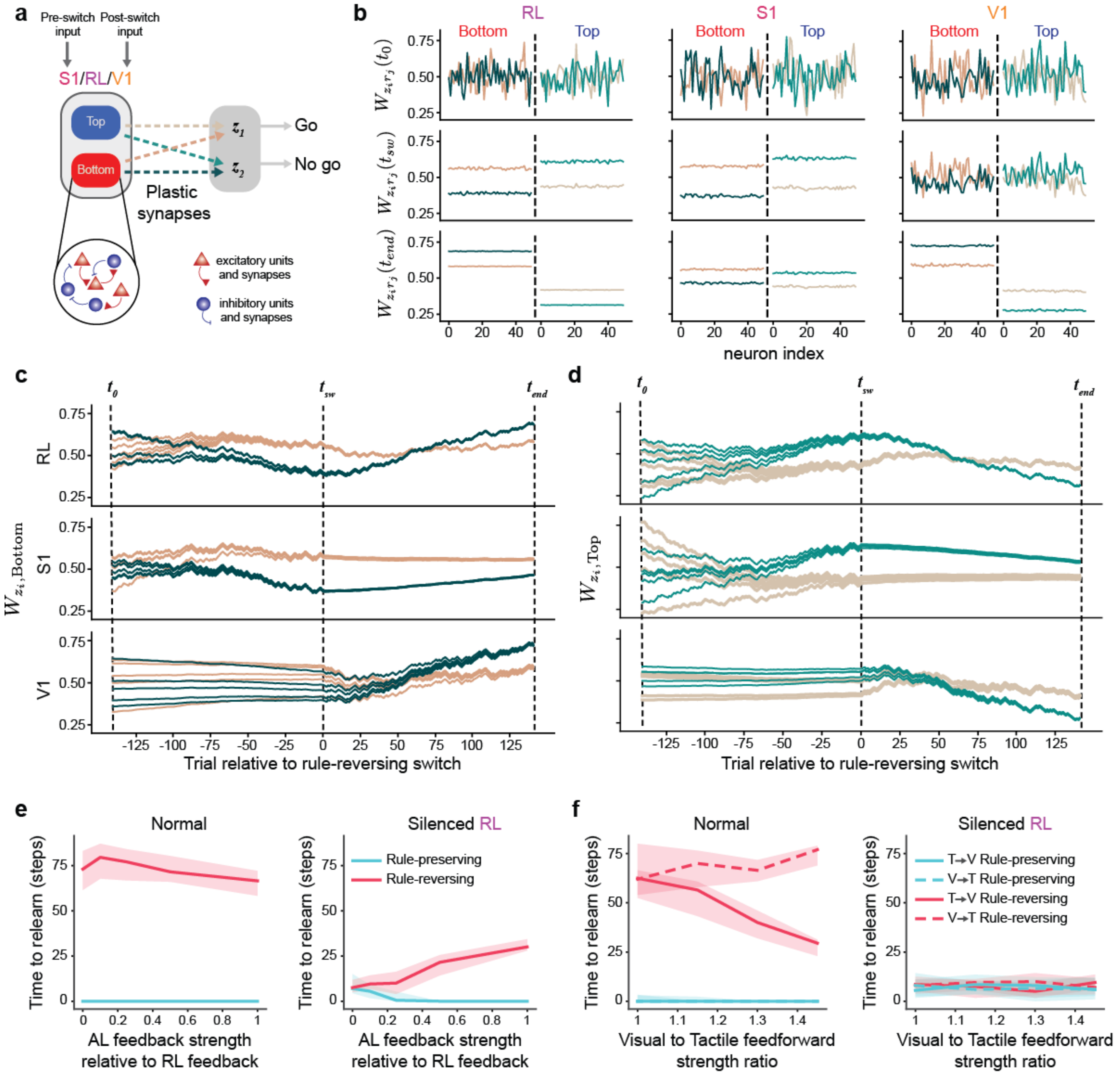

Supplementary Figure 14

Supplementary Fig. 14. Model performance on a rule-reversing modality switch.

a, Model schematic. The model consists of three networks for areas S1, V1, and RL (here only one network is shown for simplicity) that comprised excitatory and inhibitory units tuned to either bottom or top stimuli. Dashed lines denote plastic synapses, and their colors are consistent throughout the figure. The excitatory units in all networks project to the two decision variables z_1 and z_2. Area AL is included in the model but lacks direct projections to the decision area. S1 was initially stimulated, and Bottom stimuli mapped to “Go” action. After the switch, V1 was stimulated, and Top stimuli were mapped to “Go” action. **b,** Weights shown for different time points (corresponding to the dashed lines in panels c and d as a function of the neuronal index. $W_{z_i,r_j}(t)$ denotes the weights from sensory neuron j to decision variable i at time t. t_0: initial timepoint; t_{sw}: time of the switch; t_{end}: final timepoint after a rule-reversing switch. At the time of the switch (t_{sw}), all regions have the same organization of weights such that the correct output is produced (including in V1, due to the reverberation of network dynamics). After the switch, areas RL and V1 have reorganized to drive the correct output. **c,** Subsample of weights from the Bottom-preferring units to z_1 (brown) and z_2 (teal) as a function of time in a simulation. **d,** Same as in panel c for Top-preferring units. **e,** Steps required to relearn the task following a rule-preserving (blue) or rule-reversing (red) modality switch, shown as a function of AL feedback strength in the normal network (left) and in the model with RL silenced (right). **f,** Same as panel e, shown as a function of the visual-to-tactile feedforward connection strength ratio. The comparison includes modality switches from the tactile task to the visual task (solid lines) and from the visual task to the tactile task (dashed lines). Notably, the ratio reported in Supplementary Fig. 8k was estimated to be approximately 1.3. For simplicity, these simulations were conducted without including area AL.

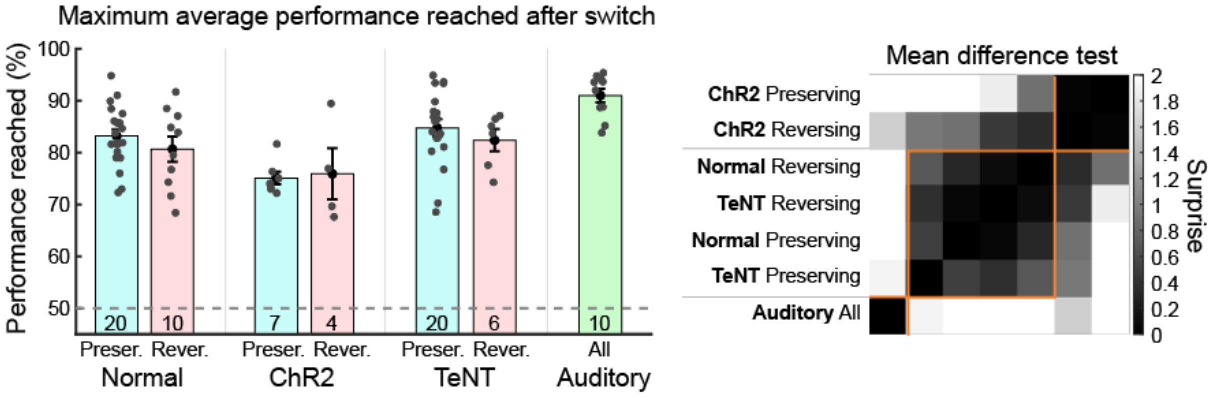

Supplementary Figure 15

Supplementary Fig. 15. Maximum average performance reached following modality switch across different conditions.

Left: Maximum average performance reached in the discrimination task following the modality switch. Mice numbers are indicated for each group at the bottom of the bar. Mice that did not reach the expert criteria in Figure 8F are discarded from this plot. Error bars: S.E.M. Right: Surprise matrix computed from pairwise unpaired two-sided t-test between conditions. A hierarchical clustering based on cosine similarity was used to group conditions based on surprise values. Mice were clustered with respect to the maximum performance reached based on the nature of sensory stimulation used after switch (visual, auditory or optogenetic).
