# Supplementary material for: Cortical circuits for cross-modal generalization
Source: Nat Commun. 2025 May 26;16:4230. doi: 10.1038/s41467-025-59342-9 (PMC12106601; doi:10.1038/s41467-025-59342-9)
Supplement: Supplementary file 2 — Reporting Summary [file 41467_2025_59342_MOESM2_ESM.pdf]

nature portfolio

Corresponding author(s): Sami El-Boustani

Last updated by author(s): Apr 14, 2025

Reporting Summary

Nature Portfolio wishes to improve the reproducibility of the work that we publish. This form provides structure for consistency and transparency in reporting. For further information on Nature Portfolio policies, see our Editorial Policies and the Editorial Policy Checklist.

Statistics

For all statistical analyses, confirm that the following items are present in the figure legend, table legend, main text, or Methods section.

n/a	Confirmed	
☐	☒	The exact sample size (*n*) for each experimental group/condition, given as a discrete number and unit of measurement
☒	☐	A statement on whether measurements were taken from distinct samples or whether the same sample was measured repeatedly
☐	☒	The statistical test(s) used AND whether they are one- or two-sided<br>*Only common tests should be described solely by name; describe more complex techniques in the Methods section.*
☐	☒	A description of all covariates tested
☐	☒	A description of any assumptions or corrections, such as tests of normality and adjustment for multiple comparisons
☐	☒	A full description of the statistical parameters including central tendency (e.g. means) or other basic estimates (e.g. regression coefficient) AND variation (e.g. standard deviation) or associated estimates of uncertainty (e.g. confidence intervals)
☐	☒	For null hypothesis testing, the test statistic (e.g. *F*, *t*, *r*) with confidence intervals, effect sizes, degrees of freedom and *P* value noted<br>*Give P values as exact values whenever suitable.*
☐	☒	For Bayesian analysis, information on the choice of priors and Markov chain Monte Carlo settings
☒	☐	For hierarchical and complex designs, identification of the appropriate level for tests and full reporting of outcomes
☒	☐	Estimates of effect sizes (e.g. Cohen's *d*, Pearson's *r*), indicating how they were calculated

Our web collection on statistics for biologists contains articles on many of the points above.

Software and code

Policy information about availability of computer code

Data collection	Matlab, ScanImage, Psychtoolbox, custom code.
Data analysis	Matlab, Suite2p, ImageJ, Python, custom code.

For manuscripts utilizing custom algorithms or software that are central to the research but not yet described in published literature, software must be made available to editors and reviewers. We strongly encourage code deposition in a community repository (e.g. GitHub). See the Nature Portfolio guidelines for submitting code & software for further information.

Data

Policy information about availability of data

All manuscripts must include a data availability statement. This statement should provide the following information, where applicable:

- Accession codes, unique identifiers, or web links for publicly available datasets
- A description of any restrictions on data availability
- For clinical datasets or third party data, please ensure that the statement adheres to our policy

The dataset used in this study is freely accessible on Zenodo at https://doi.org/10.5281/zenodo.14712478.

Research involving human participants, their data, or biological material

Policy information about studies with human participants or human data. See also policy information about sex, gender (identity/presentation), and sexual orientation and race, ethnicity and racism.

Reporting on sex and gender	n/a
Reporting on race, ethnicity, or other socially relevant groupings	n/a
Population characteristics	n/a
Recruitment	n/a
Ethics oversight	n/a

Note that full information on the approval of the study protocol must also be provided in the manuscript.

Field-specific reporting

Please select the one below that is the best fit for your research. If you are not sure, read the appropriate sections before making your selection.

☒ Life sciences ☐ Behavioural & social sciences ☐ Ecological, evolutionary & environmental sciences

For a reference copy of the document with all sections, see nature.com/documents/nr-reporting-summary-flat.pdf

Life sciences study design

All studies must disclose on these points even when the disclosure is negative.

Sample size	For the behavioral experiments, the sample size was set at a minimum of five subjects per experimental condition. This decision was made after a thorough consideration of ethical implications (3Rs principles), technical constraints, and the complexity of our experimental design. Even with this relatively modest sample size, the multitude of behavioral conditions we tested led us to include approximately 100 mice in our study. Current neuroscience literature indicates that meaningful and robust behavioral phenomena, such as those described in our work, can be discerned within this sample range, which is commonly employed in similar types of studies. For the functional imaging and anatomical analyses, the sample size was larger. This increase was feasible due to the absence of the combinatorial explosion of conditions (i.e. “direction” of the transfer x starting Go stimulus etc...) we faced in the design of the behavioral experiment (enabling us to collect a single neuron dataset of unprecedented size: tens of thousand of neurons across 25 animals).
Data exclusions	Data exclusions were implemented in instances where animals developed health issues during the experiment, which precluded the completion of the full data collection protocol. This measure was in accordance with our ethical obligation to ensure animal welfare. In addition, our experimental design necessitated that only subjects with optimal conditions for imaging or optogenetic stimulation were included in the final dataset. This included the quality of the cranial window preparation and the adequate expression of genetically encoded indicators and actuators crucial for the study. These exclusion criteria were established a priori to ensure the integrity and reliability of our experimental results.
Replication	While our results have not yet been replicated by an external laboratory (owing to their novel nature) internal replication is at the core of our experimental design. Our study's methodology involves testing the key behavioral phenomenon across a wide range of conditions and varying aspects of the original design. This allows us to observe the phenomenon from multiple perspectives, enhancing the reliability of our findings. By subjecting our hypotheses to these rigorous internal checks and controls, we believe we are upholding the highest standards of reproducibility. Furthermore, we have meticulously documented in our manuscript all necessary details to facilitate replication. As our findings become more widely recognized, we not only anticipate but encourage independent external verification to reinforce the confidence of the scientific community in our work.
Randomization	Our study, spanning four years, required the collection of various datasets that form the basis of our manuscript. During this period, mouse allocation to the experimental groups was randomized. This randomization was carried out across distinct experiment and mouse cohorts active at any given time. This approach ensured that each dataset was obtained from a representative sample of the population, thereby minimizing selection bias and ensuring that the results are generalizable.
Blinding	In the context of our study's complex animal experiments, which necessitated specific care and experimental interventions tailored to each animal based on their assigned condition, blinding of the investigators to group allocation during data collection and analysis was not feasible. Despite this, rigorous measures were implemented to maintain consistent treatment across all groups. Standardized protocols for animal handling, experimental procedures, and data recording were strictly followed to ensure that every mouse, regardless of its group, received identical care and attention. These protocols were designed to minimize any potential bias and uphold the objectivity of the data collected.

Reporting for specific materials, systems and methods

We require information from authors about some types of materials, experimental systems and methods used in many studies. Here, indicate whether each material, system or method listed is relevant to your study. If you are not sure if a list item applies to your research, read the appropriate section before selecting a response.

Materials & experimental systems

n/a	Involved in the study
☐	☒ Antibodies
☒	☐ Eukaryotic cell lines
☒	☐ Palaeontology and archaeology
☐	☒ Animals and other organisms
☒	☐ Clinical data
☒	☐ Dual use research of concern
☒	☐ Plants

Methods

n/a	Involved in the study
☒	☐ ChIP-seq
☒	☐ Flow cytometry
☒	☐ MRI-based neuroimaging

Antibodies

Antibodies used

Primary antibody: Rat anti-muscarinic acetylcholine receptor M2 (M2 AChR), Sigma-Aldrich, reference MAB367, used at a dilution of 1:500. Secondary antibodies: Donkey anti-rat Alexa Fluor 488, Invitrogen, catalog #A21208, and Goat anti-rat Cy5, Invitrogen, catalog #A10525, both used at a dilution of 1:500.

Validation

The primary and secondary antibodies were validated by the suppliers. Manufacturer's validation and relevant literature citations are available on the Sigma-Aldrich and Invitrogen websites. In further evidence for the effectiveness of our immunostaining protocol, the staining patterns we observed are fully consistent with a large body of existing literature.

Animals and other research organisms

Policy information about studies involving animals; ARRIVE guidelines recommended for reporting animal research, and Sex and Gender in Research

Laboratory animals

For the experiments reported in this manuscript we used C57BL/6J wild-type mice, Ai148-D x Rasgrf2-2A-dCre crosses, and Ai32 transgenic mice, including both males and females aged 2–5 months. Animals were housed under a 12/12-h light/dark cycle with ad libitum access to food and water except when in regime of water restriction as needed for behavioral training. The ambient temperature in the animal facility was 23 °C and the relative humidity was maintained around 50%.

Wild animals

No wild animals were involved in the study.

Reporting on sex

Both male and female mice were included in the study. This decision was based on several considerations. First, existing literature does not suggest significant sex differences in the cortical circuits we investigated, which informed our expectation of similar outcomes across sexes. Second, in adherence to the 3R principles, we aimed to limit the number of animals used without compromising the scientific integrity of our study. Lastly, logistical aspects of animal housing played a role: maintaining same-sex cages was necessary to manage breeding and prevent distress due to isolation. Data disaggregated by sex was not explicitly collected as prior data indicated no substantial sex-based variances in the primary measures of our study.

Field-collected samples

No field collected samples were involved in the study.

Ethics oversight

All procedures described in the manuscript were in compliance with the Institutional Animal Care and Use Committee of the University of Geneva and received approval from the Geneva cantonal authorities (authorization GE258B).

Note that full information on the approval of the study protocol must also be provided in the manuscript.

Plants

Seed stocks

n/a

Novel plant genotypes

n/a

Authentication

n/a
